# Spatiotemporal Dysregulation of Neuron–Glia Related Genes and Pro-/Anti-Inflammatory miRNAs in the *5xFAD* Mouse Model of Alzheimer’s Disease

**DOI:** 10.3390/ijms25179475

**Published:** 2024-08-31

**Authors:** Marta Ianni, Miriam Corraliza-Gomez, Tiago Costa-Coelho, Mafalda Ferreira-Manso, Sara Inteiro-Oliveira, Nuno Alemãn-Serrano, Ana M. Sebastião, Gonçalo Garcia, Maria José Diógenes, Dora Brites

**Affiliations:** 1 Instituto de Investigação do Medicamento (iMed.ULisboa), Faculdade de Farmácia da Universidade de Lisboa, 1649-003 Lisboa, Portugal; marta.ianni@studenti.unicam.it (M.I.); miriam.corraliza@gm.uca.es (M.C.-G.); tcoelho@medicina.ulisboa.pt (T.C.-C.); mafalda.manso@medicina.ulisboa.pt (M.F.-M.); ggarcia@campus.ul.pt (G.G.); 2 Dipartimento di Scienze della Vita, Università degli Studi di Trieste, 34127 Trieste, Italy; 3 Division of Physiology, School of Medicine, Universidad de Cadiz, 11003 Cadiz, Spain; 4 Instituto de Investigación e Innovación Biomédica de Cadiz (INIBICA), 11003 Cadiz, Spain; 5 Instituto de Farmacologia e Neurociências, Faculdade de Medicina da Universidade de Lisboa, 1649-028 Lisboa, Portugal; sara86oliveira@medicina.ulisboa.pt (S.I.-O.); nuno.aleman@campus.ul.pt (N.A.-S.); anaseb@medicina.ulisboa.pt (A.M.S.); diogenes@medicina.ulisboa.pt (M.J.D.); 6 Instituto de Medicina Molecular João Lobo Antunes, Faculdade de Medicina da Universidade de Lisboa, 1649-028 Lisboa, Portugal; 7 ULS Santa Maria, Centro Hospitalar Universitário Lisboa Norte, Centro Académico de Medicina de Lisboa, 1649-028 Lisboa, Portugal; 8 Department of Pharmaceutical Sciences and Medicines, Faculdade de Farmácia da Universidade de Lisboa, 1649-003 Lisboa, Portugal

**Keywords:** Alzheimer’s disease, *5xFAD* mouse model, hippocampus, microRNAs, neurodegeneration, neuroimmune genes, neuroinflammation, prefrontal cortex

## Abstract

Alzheimer’s disease (AD), the leading cause of dementia, is a multifactorial disease influenced by aging, genetics, and environmental factors. miRNAs are crucial regulators of gene expression and play significant roles in AD onset and progression. This exploratory study analyzed the expression levels of 28 genes and 5 miRNAs (miR-124-3p, miR-125b-5p, miR-21-5p, miR-146a-5p, and miR-155-5p) related to AD pathology and neuroimmune responses using RT-qPCR. Analyses were conducted in the prefrontal cortex (PFC) and the hippocampus (HPC) of the *5xFAD* mouse AD model at 6 and 9 months old. Data highlighted upregulated genes encoding for glial fibrillary acidic protein (*Gfap*), triggering receptor expressed on myeloid cells (*Trem2*) and cystatin F (*Cst7*), in the *5xFAD* mice at both regions and ages highlighting their roles as critical disease players and potential biomarkers. Overexpression of genes encoding for CCAAT enhancer-binding protein alpha (*Cebpa*) and myelin proteolipid protein (*Plp*) in the PFC, as well as for BCL2 apoptosis regulator (*Bcl2*) and purinergic receptor P2Y12 (*P2yr12*) in the HPC, together with upregulated microRNA(miR)-146a-5p in the PFC, prevailed in 9-month-old animals. miR-155 positively correlated with miR-146a and miR-21 in the PFC, and miR-125b positively correlated with miR-155, miR-21, while miR-146a in the HPC. Correlations between genes and miRNAs were dynamic, varying by genotype, region, and age, suggesting an intricate, disease-modulated interaction between miRNAs and target pathways. These findings contribute to our understanding of miRNAs as therapeutic targets for AD, given their multifaceted effects on neurons and glial cells.

## 1. Introduction

Alzheimer’s disease (AD) is a complex neurodegenerative disease and the leading cause of dementia in the elderly population, affecting more than 50 million people worldwide. A very small number of individuals reveal young-onset AD starting before age 65, representing up to 10% of all cases [1]. Currently, treatments for AD, such as memantine, an NMDA receptor antagonist, and cholinesterase inhibitors like donepezil, do not stop the cognitive decline and death of brain cells [2]. Fortunately, there are hope in new therapies for AD, recently approved by the FDA, based on monoclonal antibodies for the amyloid β (Aβ) peptide, though only a small percentage of older adults with early cognitive impairment have been considered eligible [3]. Aβ plaques and the intracellular neurofibrillary tangles (NFTs), composed of abnormally phosphorylated tau, are the classical hallmarks of AD and the major hypothesis recognized as associated with the progressive cognitive decline observed in patients [2,4]. The dominant risk factors for AD are advanced age and the *APOE4* genetic variant [5,6,7], leading to the premise that aging, in concert with an intricate interaction between environmental and genetic risk factors, are crucial triggers of the disease [8,9]. Around 95% of AD cases are of sporadic origin (sAD), while the remainder are hereditary cases, known as familial AD (fAD). The majority of fAD mutations involve the amyloid precursor protein (*APP*) cleavage sites for β- and γ-secretase and the subunits of the γ-secretase complex, presenilins (*PSEN1* and *PSEN2*) [10]. Recently, the concept of neurodegeneration in AD has expanded to include other non-classical alterations, such as neuroinflammation, cytoskeletal abnormalities, alterations in mitochondrial dynamics, and loss of brain homeostasis [11]. Synaptic dysfunction and chronic inflammation, involving dysregulated cellular interactions and complex signaling pathways, have been proposed as crucial players in AD [12,13,14]. This inflammation may contribute to the onset of AD or exacerbate amyloid and tau pathology [15]. In addition to the amyloid cascade and the inflammation hypothesis, we must also consider the vascular and infection hypotheses as contributing to the pathogenesis of AD [7]. Therefore, a multifaceted approach will be crucial in preventing and managing this complex disease. 

Among the various inflammatory mediators, microRNAs (miRNAs), a class of small non-coding RNAs recognized as key regulators in many essential biological processes, have emerged as important players in the onset and progression of AD pathogenesis [16]. One miRNA can target numerous genes, and one gene can be targeted by multiple miRNAs. Of note, miRNAs are particularly abundant in the central nervous system (CNS), where each cell type has unique miRNA profiles. While the dysregulation of some miRNAs may be the same between regions, in other cases it differs [17]. Elucidation of the mechanisms by which miRNAs and their multiple targets are temporally and spatially regulated or affected in the AD patient’s brain will be crucial to identifying miRNAs as biomarkers, their interrelated pathways, and those that should be used as therapeutic targets. Blocking or enhancing inflammatory signaling pathways and associated miRNAs may constitute effective strategies to prevent, or even stop, neurodegeneration due to neuronal and glial cell malfunctions [18]. However, the spatiotemporal differences of genes related to neuroimmune dysregulation and associated miRNAs have been scarcely explored in AD patients and mouse models. One of the most widely used amyloid-based models is the *5xFAD* mouse [19], which exhibits overexpression of *APP* and *PSEN1* containing five familial AD mutations: [*APP K670N/M671L* (Swedish), *APP I716V* (Florida), *APP V717I* (London), as well as the *PSEN1 M146L* and the *PSEN1 L286V*] [20,21]. The *5xFAD* transgenic mouse model recapitulates many AD-related phenotypes and manifests early disease onset, with marked age-dependent progression. Amyloid plaques and glial activation begin early, around 2 months of age, and increase rapidly with age, with plaques found throughout the hippocampus and cortex by 6 months [19]. Synaptic degeneration begins at 4 months of age, while neuronal loss occurs in brain regions with greater amyloidosis by 6 months. The *5xFAD* mice show a range of cognitive and motor deficits, with spatial working memory deficits starting at 5 months of age. 

Based on previous studies from our group, we decided in this study to assess key inflammatory genes and miRNAs known to be dysregulated in different AD models. We tested the hypothesis that inflammation-associated miRNAs (inflamma-miRNAs), such as miR-124, miR-21, miR-125b, miR-146a, and miR-155 [22,23,24,25,26], may be involved in the spatiotemporal progression of AD by impairing the intercellular crosstalk between neurons and glia in the *5xFAD* mice. miR-124 is the most predominant in the CNS, almost exclusively in neurons [27,28], and has critical nervous and immune functions [29]. Although still a matter of controversy, miR-124 has been associated with neuroprotective mechanisms [30,31,32]. In what concerns miR-21, it has been reported to have an anti-inflammatory role, to be upregulated in several AD models, and to exert protective functions in dysfunctional neurons, astrocytes, and microglia [23,33,34,35]. Overexpression of miR-125b has been shown to increase pro-inflammatory cytokines and tau hyperphosphorylation and to be associated with cognitive deficits [16,29], thus contributing to neuroinflammation and AD pathogenicity. miR-146a has been found to be upregulated in AD and associated with compensatory mechanisms toward pathological inflammation [36], or to favor tau hyperphosphorylation and the progression of mild cognitive impairment to AD [37,38]. Finally, miR-155, which has been found to increase in AD patients and mouse models, is critical for normal immune function and has been shown to regulate microglia internalization of Aβ and synaptic pruning [39,40,41]. Neuroimaging studies demonstrated that AD risk alleles have a crucial role in cortical and hippocampal morphometries [42], and alterations in AD patients and models have been mostly described in these two regions [43,44,45]. Therefore, the evaluation of hit inflammatory miRNAs and genes will be assessed in the prefrontal cortex (PFC) and the hippocampus (HPC) of 6- and 9-month-old *5xFAD* mice.

The present exploratory study proposes the following: (1) age is the main source of variation in the expression of a set of genes related to the onset and progression of AD; (2) *Gfap* (gene that encodes for the glial fibrillary acidic protein), *Cst7* (gene that encodes for Cystatin F), and *Trem2* (gene that encodes for triggering receptor expressed on myeloid cells 2) are the most promising biomarkers in both PFC and HPC in *5xFAD* mice; (3) dysregulation of inflammation-associated miRNAs and genes is more noticeable at 9 than at 6 months of age in *5xFAD* mice; (4) correlations between genes and miRNA expression levels support dynamic specificity and depend on genotype, age, and region. These findings should be considered whenever therapies targeting AD are developed or tested.

## 2. Results

### 2.1. Age-Dependent Gene and miRNA Expression Variations Predominate over Genotype and Brain Region Differences in 5xFAD Mice

**Figure 1:**
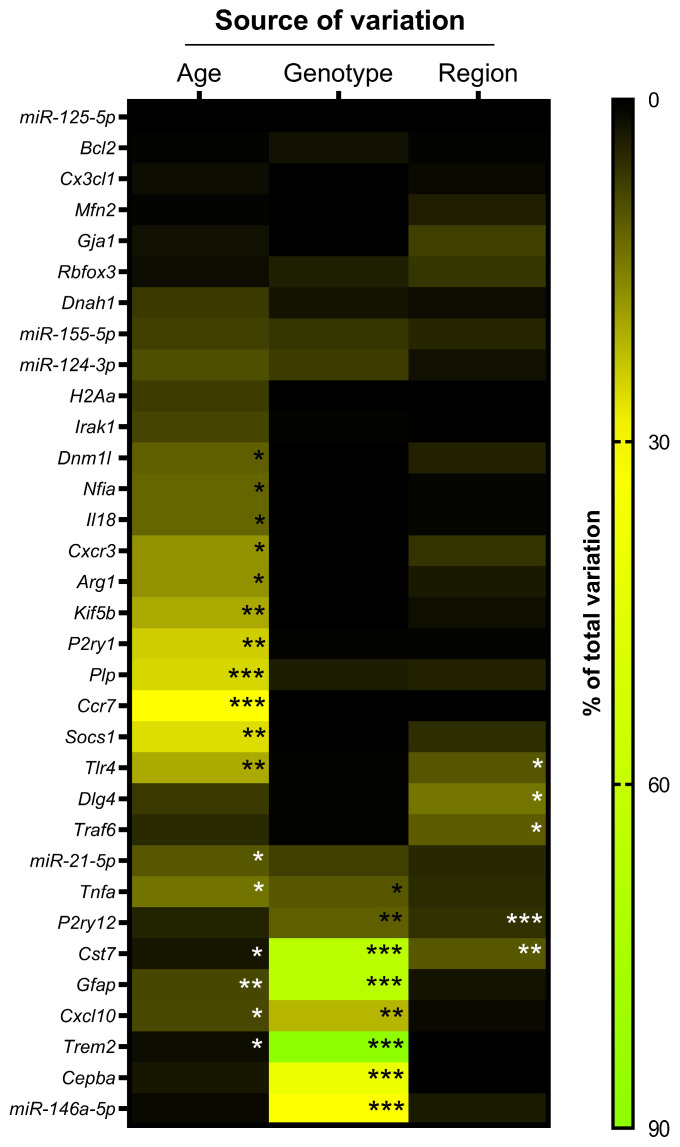
Heatmap displaying the major individual sources of variation in each of our pre-selected miRNAs and genes, by RT-qPCR analysis. Results were obtained by a three-way ANOVA (α = 0.05), with Šídák’s multiple comparisons test for the factors: age (6 vs. 9 months), genotype (*WT* vs. *5xFAD*), and region (hippocampus vs. prefrontal cortex). * *p* < 0.05, ** *p* < 0.01 and *** *p* < 0.001.

To address how age (6 vs. 9 months), genotype (wild-type, *WT* vs. *5xFAD*), and brain region (HPC vs. PFC) impacted inflammatory miRNA/gene profiling, we performed RT-qPCR analysis and three-way ANOVA. As depicted in Figure 1, age was the primary source of variation, significantly affecting 17 out of 33 significant miRNAs/genes when comparing the expression levels at 6 vs. 9 months old. Most of them are related to neuroinflammatory mediators, involving both microglia and astrocytes, while miR-21-5p showed to be the most affected by the age factor in our miRNA selection. To highlight the age-dependent effects on the axonal anterograde transport (kinesin family member 5B, *Kif5b* gene), master regulator of mitochondrial fission dynamin-related protein 1 (Drp1 encoded by the *Dnm1l* gene) and myelin proteolipid protein (encoded by the *Plp* gene).

Considering the genotype (*WT* vs. *5xFAD*), we noticed that it markedly contributed to the variations observed in a cluster of 8 miRNAs/genes, many associated with inflammation, including the CCAAT enhancer-binding protein alpha (*Cebpa*). Although affecting a lower number of genes compared to the aging effect, genotype presented an average contribution of 65%, including miR-146a, which proved to be the most affected genotype-dependent inflammation-associated miRNA.

When we studied the differences in genes/miRNAs between the PFC and the HPC, regional dependance was the least determining source of variation (below 15%) and did not influence any miRNA. Among the three sources considered here, the postsynaptic density protein 95 (Psd95, encoded by the *Dlg4* gene) and the TNF receptor-associated factor 6 (*Traf6*), an important regulator of immune homeostasis, showed to be exclusively affected by the brain region.

These results indicate that *Cst7* is the only gene (from our selection) that is simultaneously significantly affected by the three factors (age, genotype, and region). Nevertheless, the main source of variation was, by far, the genotype (65.7%, *p* < 0.001), followed by region (10.3%, *p* < 0.001) and, with much less relevance, age (2.5%, *p* < 0.05). Regarding a subset of genes recognized as glial markers, those affected by two sources of variation were Toll-like receptor 4 (*Tlr4*) for age and region, *P2ry12* for genotype and region, and *Gfap*, The C-X-C motif chemokine ligand 10 (*Cxcl10*), and *Trem2* for age and genotype.

### 2.2. The 5xFAD Mice Overexpress Genes Whose Dysregulation Depends on Age and Region

Genetic background was found to influence the proteome in the *5xFAD* mouse model [46], and genes related to detrimental and neuroprotective mechanisms were suggested to play a key role in 4- and 9-month-old animals [47]. Moreover, impaired spatial memory in the Morris water maze was reported in 6-month-old mice [48], while impaired learning was noticed in 9-month-old animals [49]. For this study, we selected a set of genes linked to the process of initiation and resolution of inflammatory conditions, including those related to microglia activation, astrocyte reactivity, and immune-associated myelin aberrancies, as well as to oxidative stress and neurodegeneration, based on our and other previous studies [23,26,50,51,52,53,54,55].

**Figure 2:**
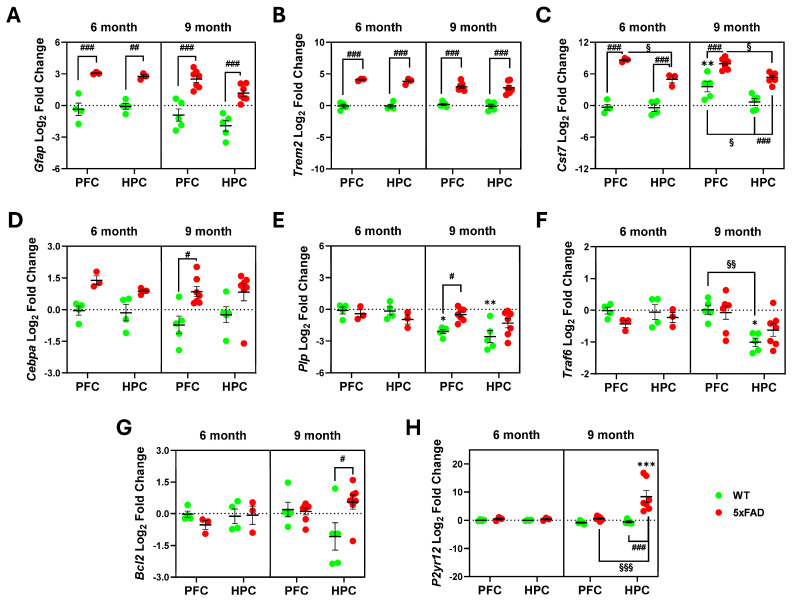
Significant differences in gene expression profiles in the prefrontal cortex (PFC) and hippocampus (HPC) of *5xFAD* mice, at 6 and 9 months of age assessed by RT-qPCR analysis. Upregulation of *Gfap* (**A**), *Trem2* (**B**), and *Cst7* (**C**) was revealed to be the most consistent markers, as they are also not dependent on age or brain region. *Cebpa* (**D**) and *Plp* (**E**) were shown to increase with aging in the PFC of *5xFAD* mice. *Traf6* (**F**) showed regional-dependent differences only in control mice, whereas the *Bcl2* (**G**) and P2yr12 (**H**) genes exhibited elevated levels in the HPC compared to the PFC. Three-way ANOVA (α = 0.05) with Šídák’s multiple comparisons test for the following factors: age (6 vs. 9 months), genotype (*WT* vs. *5xFAD*), and region (HPC vs. PFC). Statistical significance: Age comparisons: * *p* < 0.05, ** *p* < 0.01 and *** *p* < 0.001; genotype comparisons ^#^ *p* < 0.05, ^##^ *p* < 0.01 and ^###^ *p* < 0.001; region comparisons: ^§^ *p* < 0.05, ^§§^ *p* < 0.01 and ^§§§^ *p* < 0.001.

Among the several genes that were assessed in 6- and 9-month-old *5xFAD* mice, *Gfap*, *Trem2*, and *Cst7* were found upregulated in the HPC and the PFC at both ages (*p* < 0.001) when compared with corresponding *WT* mice (Figure 2A–C). *Cst7* was also shown to increase with age in the PFC of *WT* mice (*p* < 0.01) and to be less upregulated in the HPC than in the PFC in the *WT* and *5xFAD* mice (*p* < 0.05). Our data highlight these genes as the most significantly affected among those studied, and they serve as key markers of glial activation and disease pathogenicity in the *5xFAD* mouse model. The PFC region-specific biomarkers *Cebpa* and *Plp* were shown to be enhanced in 9-month-old *5xFAD* mice when compared with *WT* mice **(**Figure 2D,E, *p* < 0.05). While *Plp* values were significantly lower in 9-month-old *WT* mice (PFC *p* < 0.05; HPC *p* < 0.01), curiously the same was not observed in the *5xFAD* model from 6- to 9-month-old animals. *Traf6*, that participates in many protective responses [56], showed a decrease by age in the HPC (*p* < 0.05) and from the PFC to the HPC (*p* < 0.01) in *WT* mice (Figure 2F). Similarly, the HPC region-specific genes encoding for BCL2 apoptosis regulator (*Bcl2*) and for purinergic receptor (*P2yr12*) increased only at 9 months in *5xFAD* mice (*p* < 0.05 and *p* < 0.001, Figure 2G,H, respectively), relatively to *WT* animals. Regarding other microglia-associated genes, such as arginase 1 (*Arg1*), CXC motif chemokine receptor 3 (*Cxcr3*), and the major histocompatibility complex class II coding gene (*H2Aa*), no significant differences were found in transgenic (*Tg*) vs. *WT* mice, neither regionally nor temporarily (Supplementary Table S1).

In the same way, neuron-associated *Dlg4*, *Rbfox3* (encodes NeuN), *Kif5b* (encodes Kinesin Family Member 5B), *Dnah1* (encodes dynein axonemal heavy chain 1), and *Cx3cl1* (also known as fractalkine or C-X3-C motif chemokine ligand 1, signaling through its unique receptor *Cxcr3* expressed in microglia) did not show differences. *Cxcl10* engaged in homeostasis and mainly expressed by neurons [57], remained unchanged (Supplementary Table S1), though a slight upregulation was noticed in the PFC of 9-month-old *5xFAD* mice (*p* = 0.09).

In addition to the significance seen for *Gfap*, a marker of astrocyte reactivity, we were unable to find age or spatiotemporal differences for other biomarkers of astrocyte hyperactivity in *5xFAD* mice, such as nuclear factor I A (*Nfia*), purinergic receptor P2Y1 (*P2ry1*), and gap junction protein alpha 1 (*Gja1*) that encodes connexin-43.

When we looked at central suppressors or mediators of AD-related neuroinflammation, including tumor necrosis factor alpha (*Tnfa*), interleukin-18 *(Il18*), interleukin-1 receptor-associated kinase-1 (*Irak1*), the C-C motif chemokine receptor 7 (*Ccr7*), suppressor of cytokine signaling factor 1 (*Socs1*), and *Tlr4*, no significant differences were found in terms of genotype, age, or brain region in *5xFAD* mice by three-way ANOVA (Supplementary Table S1). Note, however, that statistically significant differences were obtained by two-way ANOVA between 6 and 9 months for *Socs1*, *Ccr7*, and *Tlr4*, as indicated in the Supplementary Table S1 footer. No alterations were found in the *Dnm1l* gene that encodes for dynamin-related protein 1 (Drp1), linked to mitochondrial fission [58], or in mitofusin 2 (*Mfn2*) that regulates mitochondrial fusion and cellular metabolism [59].

In sum, our study identified genotype-, age-, and regional-associated upregulated genes in the *5xFAD* mice that should always be considered whenever pathogenicity and therapeutic strategies are to be explored.

### 2.3. miRNA Gene Ontology Indicates Common Pathways and Dysregulated Expression Levels in 5xFAD Mice with HPC and PFC Differences in 6- and 9-Month-Old Animals, but No Effective Clusters

Studies on human samples from AD patients have suggested that some specific miRNAs are dysregulated, and their alterations may contribute to the pathogenesis of the disease [16]. For the present analysis, we selected miR-124-3p, miR-125b-5p, miR-146a-5p, miR-155-5p, and miR-21-5p, as they have previously been shown to contribute to inflammatory states and to be dysregulated in AD patients and disease models [22,36,60,61,62].

**Figure 3:**
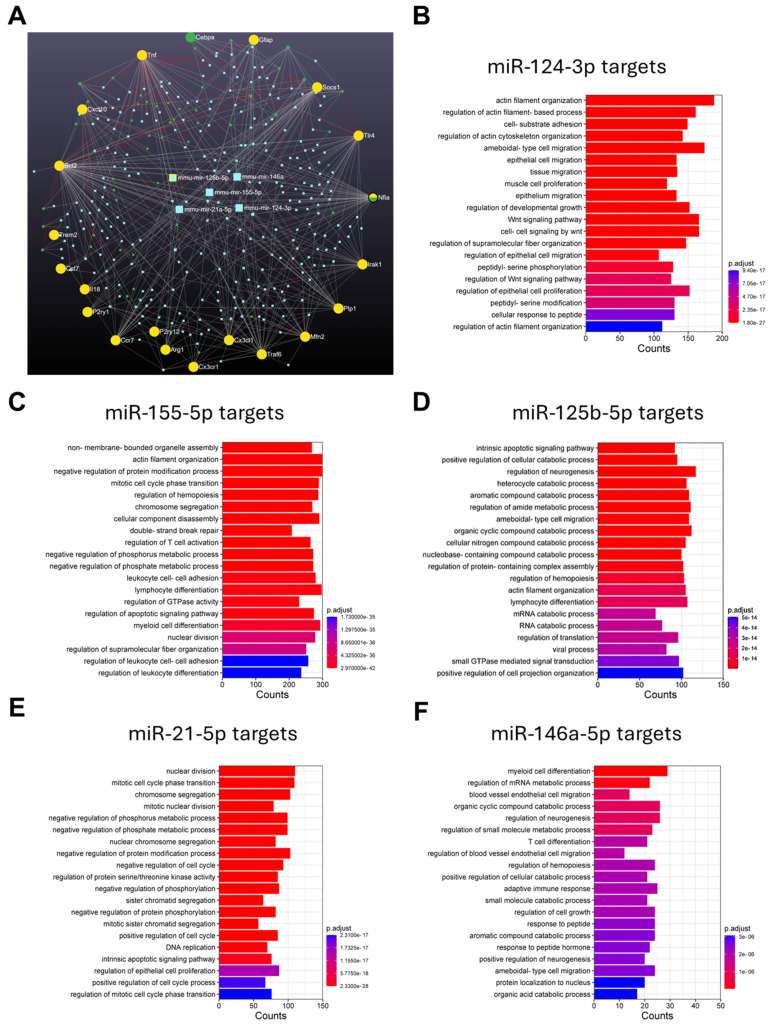
Inflamma-miRNA network and gene ontology. (**A**) miRNA–target network-map for the analyzed microRNAs and targets selected for this study. (**B**) miR-124-3p gene ontology enriched analysis. (**C**) miR-155-5p gene ontology enriched analysis. (**D**) miR-125b-5p gene ontology enriched analysis. (**E**) miR-21-5p gene ontology enriched analysis. (**F**) miR-146a-3p gene ontology enriched analysis.

We constructed a miRNA–target interaction map and table (Figure 3A and Supplementary Tables S2 and S3) to cluster the interactions between our pre-selected miRNAs and genes within a comprehensive database that included all known miRNA–target interactions. Our pre-selected miRNAs and genes presented larger nodes than other non-selected miRNAs/genes, attesting their potential interactions. Among the five miRNAs (cyan squares in the center), a total of 16 nodes were detected for miR-155-5p, followed by 9 nodes for miR-124-3p and miR-21a-5p, then 4 for miR-125b-5p, and only 2 for miR-146a-5p. Among the genes assessed (yellow/green circles), we highlight a total of 91 nodes for *Bcl2*, 56 for *Tnfa*, 47 for *Nfia*, and 36 for *Traf6* (Supplementary Table S3). This miRNA–target interaction map depicts how closely interconnected our pre-selected miRNAs and genes are, compared to all miRNA–target interactions described in the literature.

Next, a gene ontology (GO) enrichment analysis was performed to better understand which biological processes (BP) and pathways target these miRNAs. As depicted in Figure 3B, miR-124-3p was revealed to be involved in cell migration, cytoskeleton modifications, and signaling pathways. Similarly to miR-124-3p, miR-155-5p demonstrated involvement in cytoskeleton organization (Figure 3C). The highest number of common pathways was shared between miR-155-5p, miR-125b-5p and miR-21-5p (Figure 3C–E), including regulation of metabolic processes and protein modifications, as well as with the cell-cycle and DNA processes. Finally, miR-146a appeared as the most heterogenous in terms of targets, as depicted in Figure 3F.

**Figure 4:**
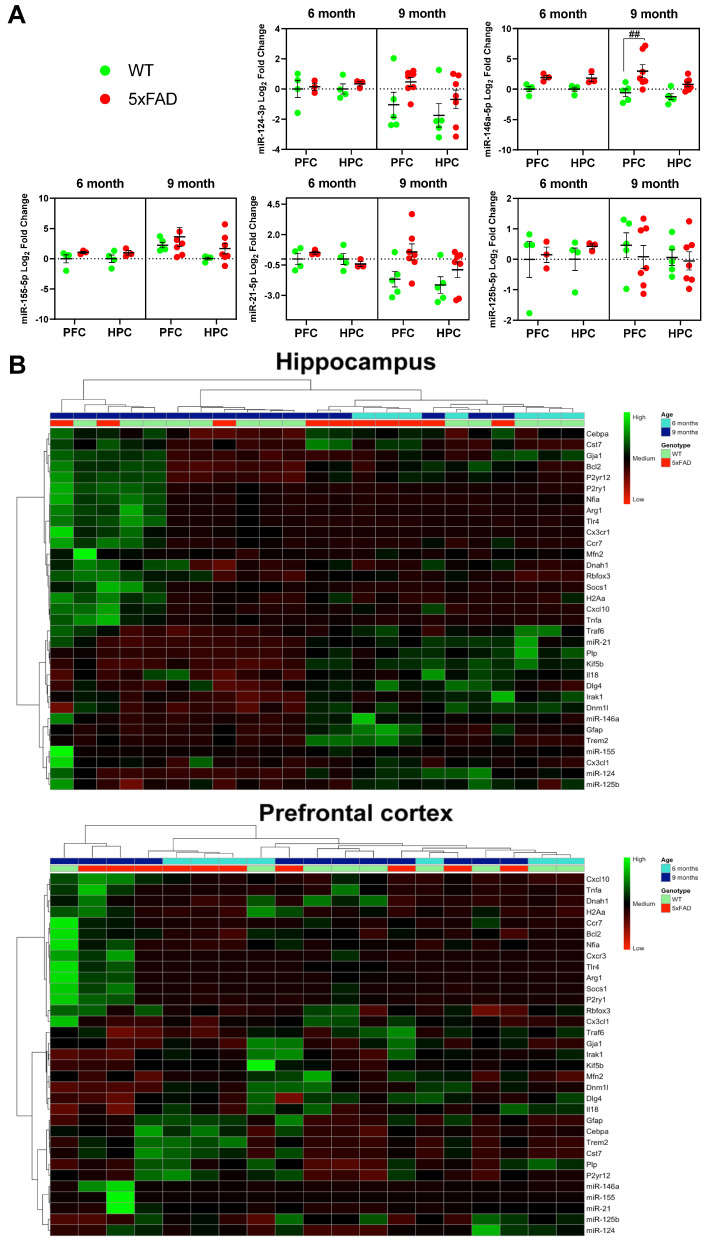
Inflamma-miRNA expression levels, heat map diagram, and cluster analysis of miRNAs and genes considering age and genotype. (**A**) Expression level of each hit inflamma-miRNA by RT-qPCR. Three-way ANOVA (α = 0.05), with Šídák’s multiple comparisons test for the following factors: age (6 vs. 9 months), genotype (*WT* vs. *5xFAD*), and region (hippocampus vs. prefrontal cortex). Age comparisons: ^##^ *p* < 0.01. (**B**) Clustered heatmap of the differentially expressed genes and miRNAs. The parameters whose expression is greater in the case group are shown in green and those smaller in red. Darker colors represent less significant values.

Thereafter, we checked the expression levels of such hit miRNAs in the HPC and the PFC of the *5xFAD* and *WT* mice, in both 6- and 9-month-old animals. We aimed to identify early and late temporal and regional biomarkers, as age is one of the main risk factors for AD [63]. Despite the global dysregulation of the 5 selected miRNAs, only significant upregulation for miR-146a-5p was found in 9-month-old PFC samples from *5xFAD* mice (Figure 4A). Interestingly, the differences did not follow a specific pattern, i.e., age or genotype, but rather evidenced to be heterogenous for each miRNA expression level.

Increased statistical trends were noticed for miR-124-3p (*p* = 0.09) and miR-21-5p (*p* = 0.12) between *5xFAD* mice and *WT* animals at 6- and 9-months-old, validating the influence of the factor age on miRNA expression profiles. In general, variation between samples was more evident in 9-month-old samples compared to their expression levels at 6-months-old. From this, we decided to perform a clustered heatmap to better visualize any possible cluster due to age or genotype. This analysis provided a graphical representation of the data set where values appear in different colors and/or shades, facilitating a visual summary of the output, which represents relationships that may exist across two variables with dendrograms to visualize clusters and accentuate similarities or differences between variables. The heatmap pointed to age as the most powerful factor in HPC compared to PFC (Figure 4B). Indeed, HPC parameters were clearly divided between 6- and 9-month-old samples, while PFC parameters were scattered. However, neither HPC nor PFC profiles showed a relevant genotype-dependent pattern. The data corroborate the existence of region-dependent aging signatures.

### 2.4. Correlations between miRNAs and Genes Implicated in Neuroinflammation and Disease Onset/Progression Are Genotype-, Region-, and Age-Dependent

**Figure 5:**
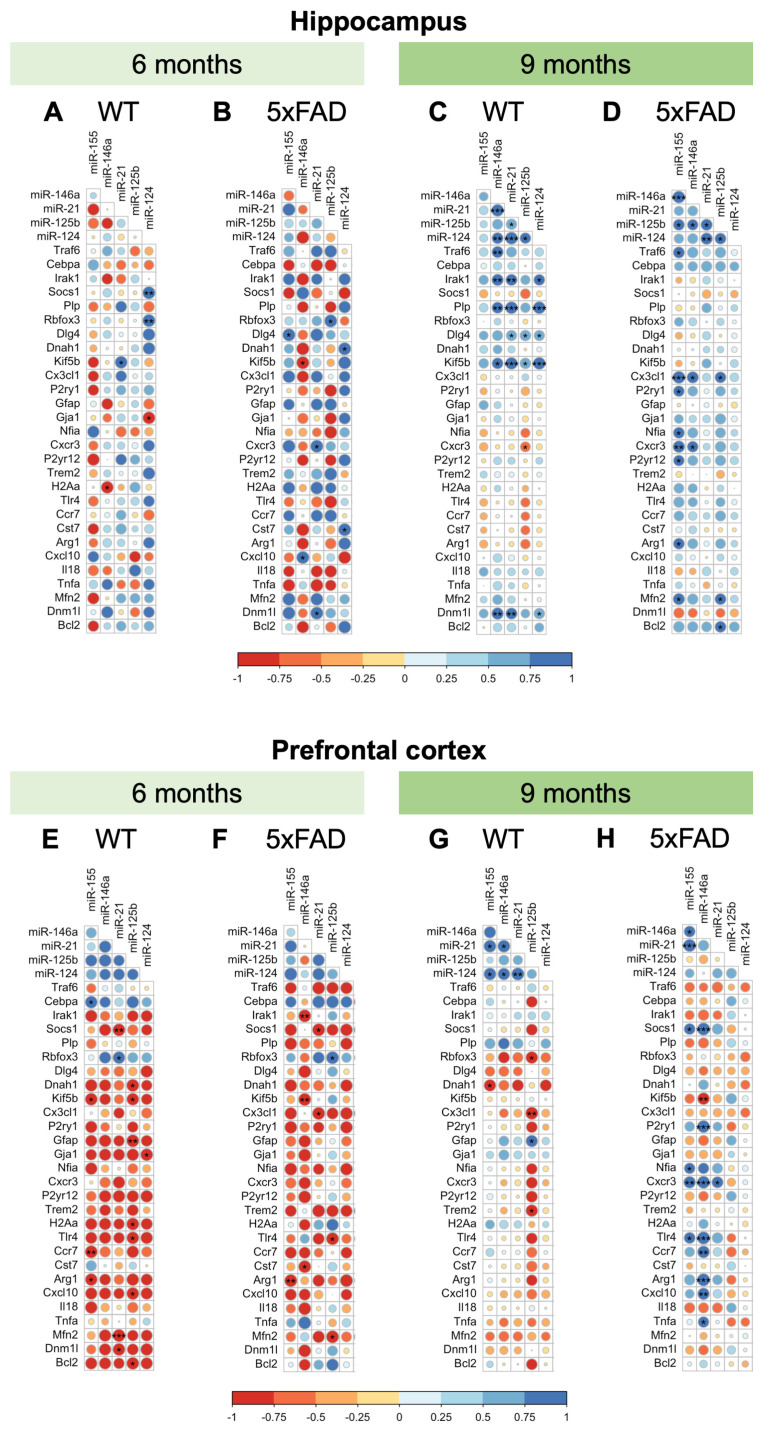
Correlation analysis between the investigated set of miRNAs and gene expression levels in the *5xFAD* mouse model of AD. The correlation matrix represents the pairwise correlations using Pearson’s correlation coefficient. Positive correlations are shown in blue and negative correlations in red. Color intensity and size of the circles are proportional to the correlation coefficient. (**A**) *WT*, 6 months hippocampus; (**B**) *5xFAD*, 6 months hippocampus; (**C**) *WT*, 9 months hippocampus; (**D**) *5xFAD*, 9 months hippocampus; (**E**) *WT*, 6 months prefrontal cortex; (**F**) *5xFAD*, 6 months prefrontal cortex; (**G**) *WT*, 9 months prefrontal cortex; (**H**) *5xFAD*, 9 months prefrontal cortex. Significance of Pearson’s correlation coefficients: * *p* < 0.05, ** *p* < 0.01 and, *** *p* < 0.001.

To clarify potential associations between miRNA dysregulation and AD-induced alterations in biological processes, we performed a correlation analysis. The overview of the bivariate analysis results revealed differential correlation patterns across the three factors studied: region, age, and genotype (Figure 5 and Supplementary Table S4). 

Within HPC genes and miRNAs (Figure 5A–D), there was a marked effect of age on both genotypes and a general shift towards positive correlations between target miRNAs and gene expression levels, suggesting that genes and miRNA interactions evolve during disease progression. At 6-months-old, no significant correlations were noticed in the HPC between miRNAs, but some association trends were clearly modulated by the genotype, such as miR-21-5p with miR-155-5p (Figure 5A,B). Gene-miRNA correlations were also sharply genotype-dependent. In *WT* mice, positive correlations were detected between miR-124-3p and *Socs1*, and *Rbfox3*, as well as miR-21-5p and *Kif5b*, while negative correlations were found between miR-124-3p and *Gja1* and miR-146a and *H2Aa* (Figure 5A). On the other hand, in the 6-month-old *5xFAD* mice *Rbfox3* positively correlated with miR-125b-5p, and *Dlg4* was correlated with miR-155-5p. Among the genes that target motor proteins in *5xFAD* mice, *Dnah1* was positively correlated with miR-124-3p and *Kif5b* negatively correlated with miR-146a-5p. In addition, miR-124-3p showed a positive correlation with *Cst7*, the most consistently upregulated gene in *5xFAD* mice (https://doi.org/10.57718/model-ad-explorer, accessed on 24 June 2024), miR-21-5p positively correlated with *Cxcr3*, and miR-146a showed a direct association with *Cxcl10* expression (Figure 5B). The data indicate that the interactions between the expression of miRNAs and genes appear to be quite complex and modulated by the disease.

In 9-month-old mice, positive correlations were detected in HPC samples between miRNAs (e.g., miR-124-3p with miR-21-5p, miR-124-3p with miR-125b-5p, and miR-125b-5p with miR-21-5p) in both genotypes, suggesting an age-mediated co-expression pattern. On the other hand, miR-146a-5p correlations with other miRNAs were genotype-dependent (positive correlations with miR-21-5p and miR-124-5p in *WT* mice, and with miR-155-5p and miR-125b-5p in *5xFAD* mice) (Figure 5C,D). Such results indicate a disease-mediated change of miR-146a-5p co-expression with the other hit miRNAs. In the 9-month-old *WT* mice (Figure 5C), some genes showed positive correlations with multiple miRNAs. Notably, miR-21-5p and miR-124-3p positively correlated with *Irak1*, *Plp*, *Dlg4*, *Kif5b*, and *Dnm1l*, indicating that these two miRNAs have a directional and concomitant influence on the regulation of these target genes, which may implicate common signaling pathways. In addition, miR-125b-5p showed positive correlations with *Dlg4 and Kif5b*, while miR-146a-5p was positively correlated with *Traf6*, *Irak1*, *Plp*, *Kif5b*, and *Dnm1l*. Conversely, the correlation pattern was totally different in age-matched *5xFAD* mice (Figure 5D). In these animals, miR-155-5p showed positive correlations with several genes, including *Traf6*, *Cx3cl1*, *P2yr1*, *Nfia*, *Cxcr3*, *P2yr12*, *Arg1*, and *Mfn2*, indicating a prominent role for this miRNA in regulating gene expression in both neurons and glial cells in the context of AD. Considering miR-146a, it positively correlated with *Cx3cl1* and *Cxcr3*. Furthermore, miR-125b-5p positively correlated with *Mfn2* and *Bcl2*, suggesting a role for this miRNA in the regulation of mitochondria-associated processes. 

In the PFC, the scenario was completely different from the HPC (Figure 5E–H). Negative correlations between the assessed miRNAs and gene expression levels prevailed over positive correlations at 6 months of age. Only two positive correlations were found in the *WT* PFC (Figure 5E), between miR-155-5p and *Cebpa*, and between miR-21 and *Rbfox3*. Indeed, miR-155-5p negatively correlated with *Kif5b*, *Ccr7*, and *Arg1*, and miR-21-5p with *Socs1*, *Mfn2*, and *Dnm1l*. Also negatively correlated were miR-125b-5p with *Dnah1*, *Kif5b*, *Gfap*, *H2Aa, Tlr4*, *Cxcl10* and *Bcl2*, as well as miR-124-3p with *Gja1*. In the *5xFAD* mice of the same age (Figure 5F), some of these correlations were maintained, including *Socs1* with miR-21-5p, *Tlr4* with miR-125b-5p, and *Arg1* with miR-155-5p. However, other correlations were disease-specific, either the positive correlation of *Rbfox3* with miR-125b-5p, or the negative correlations of miR-146a-5p with *Irak1*, *Kif5b*, and *Cst7*, as well as of miR-21-5p with *Cx3cl1*.

At the age of 9 months, positive correlations in the PFC were found for miR-124-3p with miR-155-5p, miR-146a-5p, and miR-21-5p specifically in *WT* mice (Figure 5G), and for miR-146a-5p with miR-155-5p only in *5xFAD* mice (Figure 5H). Considering correlations between miRNAs and genes, negative correlations were found for miR-155-5p with *Dnah1*, and for miR-125b-5p with *Rbfox3*, *Cx3cl1*, and *Trem2* in the *WT* PFC (Figure 5G), while the only positive correlation observed for miR-125b-5p was with *Gfap*. In 9-month-old *5xFAD* mice, the only negative correlation was identified between miR-146a and *Kif5b* in the PFC (Figure 5H). Positive correlations were found for miR-155-5p, miR-146a-5p, and miR-21-5p with *Cxcr3*, and for miR-155-5p and miR-146a-5p with *Socs1* and *Tlr4*. Moreover, miR-155-5p selectively correlated with miR-146a-5p, miR-21-5p, and *Nfia*, while miR-146a-5p selectively correlated with *Ccr7*, *P2ry1*, *Arg1*, *Cxcl10*, and *Tnfa*. 

In sum, there are several interrelationships between miRNAs and gene expression levels associated with neurodegeneration and neuroinflammation in the *5xFAD* mice, with different presentations depending on the age of the animal and brain region. These findings highlight that controversies in previous studies and the ineffectiveness of therapeutic strategies can derive from differences in patients’ genotypes, but also from the stage of the disease and the brain regions affected by AD, such as the HPC and the PFC.

## 3. Discussion

AD is a chronic neurodegenerative disorder with a countless number of potential markers and is a swiftly escalating global health problem. The multifactorial nature of AD and its pathophysiological subtypes [64] may require a tailored therapy together with additional multiple strategies, as shown by the clinical trials targeting Aβ having as much as a 99% failure rate [65,66]. Therefore, there is an urgent need for treatments that can meet the individual needs of patients, considering the different stages of AD pathogenesis to achieve better outcomes, as the global population is rapidly aging, which is a primary risk factor. Although many studies have explored the molecular changes in AD, the spatiotemporal heterogeneity of gene and miRNA alterations remains poorly understood, complicating the identification of therapeutic targets and biomarkers. Several studies have been published on the influence of miRNAs on AD onset and progression, showing their involvement in Aβ metabolism, tau phosphorylation, synaptic function, and neuroinflammation [62,67,68]. Thus, gene regulatory miRNAs have been advancing remarkably fast as promising AD stage biomarkers and potential therapeutics [69]. As several miRNAs have been found to be differentially expressed in AD [70], and multiple core genes related to AD pathogenesis across multiple brain regions have been identified [45,71,72], future investigations are required to define miRNA/gene spatiotemporal distribution and miRNA potential as disease modulators in preclinical trials. Dysregulated miRNAs, such as miR-124-3p, miR-125b-5p, miR-146a-5p, miR-155-5p, and miR-21-5p, known as inflamma-miRNAs, have been prominently featured in *in vitro* studies mimicking AD, biological fluids and biopsy specimens of AD patients [1,26,60,61,62,73,74].

A dynamic molecular network of gene regulatory elements, changing with age, was proposed to underlie age-dependent AD pathogenesis [8,75]. Studies demonstrated that a neuroinflammatory status, induced by genetic variations in CNS cells or by peripheral immune cells, begins decades before the appearance of cognitive impairment in AD patients [76,77]. The chronic persistence of such neuroinflammation leads to alterations in the immune system and cellular senescence, with cells being less responsive to insults. Pathological microglia and astrocytes [78] acquire several regional- and temporal-dependent phenotypes that contribute to the development and progression of AD [76,77,79,80]. In addition, miRNAs, as immune system and senescence regulators (mainly miR-146 and miR-21), as well as modulators of the aging process [81,82], are less explored in the context of AD temporal course and susceptibilities.

In this work, we hypothesized that inflamma-miRNAs are involved in the spatiotemporal progression of AD by targeting neuroinflammatory processes, as we showed in some previous studies [53,83,84]. To this end, we analyzed the top five inflamma-miRNA signatures along with genes involved in dysregulated neuroglial signaling, inflammation, and AD onset and progression using the PFC and the HPC samples from *5xFAD* transgenic mice and their corresponding *WT* littermates (B6SJLF1/J). The choices were as follows: miR-124, for regulating the expression of BACE1 [32] and for being proposed as a therapeutic promise for neurodegenerative disorders [85]; miR-125b-5p, for inducing tau hyperphosphorylation and cognitive deficits in AD [86]; miR-21, for inhibiting Aβ-induced apoptosis and being upregulated in AD [34]; miR-146a, for being related to cognitive deterioration and increased expression of Aβ, Taup38, and reactive oxygen species through targeting MAPK signaling [68]; and miR-155, due to its critical role in regulating memory impairment in AD rodents via engagement of neuroinflammatory mechanisms [40,87]. Considering age as a critical risk factor for AD, we examined these parameters at two symptomatic stages of disease progression, 6 and 9 months, respectively, to understand time-dependent variations in dysregulated pathways.

Of the 28 assessed core genes associated with inflammation and neurodegeneration, 17 showed to be markedly conditioned by age (from which 11 were unique), 8 with the genotype (from which 3 were unique), and 5 with the brain region (from which 2 were unique). It was previously demonstrated in *5xFAD* mice that several genes were common to the cortex and the hippocampus at 6 and 9 months, including *Cst7* and *Gfap* [47], which were also observed by us and validated the model. The upregulation of *Cst7* and *Gfap* is related to inflammation mediated by microglia and astrocytes, respectively [88].

*Cst7*, a key cystatin isoform putatively involved in immune regulation [89], was the only one that simultaneously increased across the three sources of variation considered in this study. We found that it was markedly elevated in the *5xFAD* mice as early as 6 months of age and higher in the PFC than in the HPC region. Other studies have also identified Cst upregulation in the APP/PS1 mice cortex of at 23–24 months old [90], in the PFC of AD patients [91], and in disease-associated microglia (DAM), where the upregulation also observed was found to be *Trem2*-dependent [92]. Notably, *Cst7*, by negatively regulating microglia phagocytosis, may contribute to Aβ and tau accumulation when overexpressed [93]. Interestingly, we noticed, for the first time, that its expression seems to positively correlated with miR-124-3p in the HPC but negatively correlated with miR-146a-5p in the PFC, in the case of 6-month-old *5xFAD* mice, in accordance with its region-dependent variations and immunoregulatory role. In future studies with miRNA mimics and inhibitors as therapeutic strategies, it may be interesting to explore the consequences on their expression levels according to the AD pathogenicity features.

*Gfap* is used as an early indicator of CNS injury and an identifier of astrocytes and associated reactivity [94,95]. In this study, it was markedly influenced by the genotype and age, being similarly elevated in the PFC and HPC regions of the *5xFAD* mice. GFAP is considered a potential biomarker for the early diagnosis of dementia and AD [96,97], in line with our findings. The third parameter that behaved as *Gfap* was *Trem2*, showing increased levels at 6- and 9-month-old *5xFAD* mice in both brain regions. The *Trem2* gene was suggested to be a response of microglia to elevated levels of Aβ [98] and to be associated with their enhanced phagocytosis [99]. DAM cells were shown to depend on *Trem2* in the *5xFAD* mouse at 7-month-old [100]. However, there was no investigation on regional differences, and the role of TREM2+ microglia in AD pathology is not clear. TREM2 upregulation is considered a risk factor in AD because it can affect cholesterol, myelin, and phospholipid metabolism, negatively influencing the pathogenesis of the disease at both the CNS and periphery [101] and being more injurious at the early stages [102]. Elevation in TREM2 was shown to increase peri-plaque microglial activation and neuritic plaque–tau pathology, as well as neuritic dystrophy [103]. Given that microglial function depends on the context [104] and disease stage [105], more studies are necessary to better understand the role of the DAM phenotype in AD patients with mild cognitive impairment/very mild dementia and amyloid deposition. Interestingly, we were unable to find any correlation with our set of inflamma-miRNAs either for *Gfap* or *Trem2*, though it was validated for miR-34a impact [106], a known tumor suppressor not assessed in the present study. *Cebpα* and *Plp* alterations were mainly noticed in the 9-month-old *5xFAD* mouse in the PFC region. *Cebpα*, recently indicated as a positive transcriptional regulator of disease lipid-associated microglia subpopulations [107], showed a pattern like *Trem2*, suggesting that these subpopulations are prevalent in this region. The *Plp* gene was increased in the PFC of 9-month-old *5xFAD* mice, while *WT* mice had low *Plp* levels in the PFC and HPC. In fact, decreased concentrations of PLP and white matter abnormalities were previously observed in AD cerebral cortex [54,108].

In 9-month-old *WT* mice, *Plp* correlated positively with miR-146a-5p, miR-21-5p, and miR-124-3p in HPC. *Traf6* levels, which decreased between the PFC and the HPC regions in *WT* mice at the same age, were not regionally dependent in matched *5xFAD* mice. Notably, *Traf6* positively correlated with miR-146a-5p in *WT* mice and with miR-155-5p in *5xFAD* mice in the HPC region. Although *Traf6* has been indicated as a direct target of miR-146a [109], in some circumstances such a signaling axis might work differently [110].

*Bcl2* levels were elevated in the HPC of *5xFAD* mice at 9 months and showed to positively correlate with miR-125b-5p in the same animals, but negatively in the PFC of 6-month-old *WT* mice. Since an inverse correlation was expected from previous studies supporting Bcl2 as a target of miR-125b [111], the reason why this did not happen may derive from our data spread. Bcl2 proteins were shown to have neuroprotective and anti-apoptotic functions and help to normalize dysregulated Ca^2+^ signaling (synaptoprotective) in the *5xFAD* mouse model of AD [112]. These protective effects are rather complex and deserve further studies. In postmortem AD patient samples, Bcl2 immunoreactivity was found in astrocytes, near Aβ plaques assisting cell survival, but was decreased in degenerating neurons [113].

*P2ry12* was shown to be the only genotype-dependent gene in our study, and in contrast to our results pointing to an increase of *P2ry12* in the HPC of *5xFAD* mice at 9-month-old, others found it in the cortex and at 12-month-old [114]. However, *P2ry12*, a useful marker for the identification of healthy microglia, was shown to be typically downregulated in DAM cells in AD and associated with tau rather than with Aβ pathology [115]. We only found a positive correlation between *P2yr12* and miR-155 in the HPC of 9-month-old transgenic mice, which may suggest that miR-155 modulates P2R signaling, as previously reported in dendritic cells [116]. However, we must take into consideration that such correlations may not be mutually causal since they can be due to indirect relationships between parameters.

*Tlr4* expression was not genotype-dependent but was influenced by age and brain region. Age-related variations in TLR4 expression have been identified and related to stress and persistent immune responses, exacerbating aging-related disorders [117,118]. TLR4 affects hippocampal neurogenesis and modulates hippocampus-dependent learning and memory [119,120]. *Tlr4* seems to negatively correlate with miR-125b-5p in the younger *WT* and transgenic mice and positively with miR-155-5p and miR-146a-5p in the older animals, both in PFC. Interactions of TLR4 with miR-155, miR-125b, and miR-146a were already demonstrated [121,122,123].

*Dlg4* appears to be strictly dependent on the brain region. Although we did not identify a decline in *5xFAD* mice [19] other than a slight decrease in the PFC of 9-month-old diseased mice, we obtained a positive correlation with miR-155-5p in the HPC of younger *5xFAD* animals.

Despite previous observations on neuron loss and intraneural Aβ42 accumulation in 1.5-month-old *5xFAD* mice [124], downregulation of *Rbfox3* has only been documented for 12 months in other studies [114], and we have not confirmed this. *Rbfox3* correlated with miR-125b-5p in the younger transgenic mice in both brain regions we evaluated, but not subsequently. Our study did not detect changes in *Rbfox3* expression levels, contrasting with research supporting miR-125b upregulation in AD [125]. Studies have shown that miR-125b-5p overexpression increases pro-inflammatory cytokines and induces tau hyperphosphorylation, leading to cognitive deficits [16,29], thus playing a critical role in neuroinflammation while contributing to AD [61].

Expression levels of *Tnfa* and *Cxcl10* varied with age and genotype, but only *Cxcl10* was revealed to be enhanced in the *5xFAD* mice, despite not reaching statistical significance. We noticed the presence of positive correlations between *Cxcl10* and miR-146a in both the HPC of younger and in the PFC of older *5xFAD* mice. *Tnfa* also seemed to correlate with miR-146a-5p in the PFC of 9-month-old *5xFAD* mice. Interestingly, miR-146a, another pivotal neuroimmune miRNA in the CNS and peripheral immune cells [126], was upregulated in AD patients’ brains and linked to tau hyperphosphorylation by suppressing ROCK1 [37].

In 9-month-old *5xFAD* mice, miR-146a-5p was upregulated in the PFC and positively correlated with miR-155-5p. Remarkably, miR-146a-5p directly correlated with miR-155-5p in the HPC in the same group of mice. This upregulation of miR-146a-5p may serve as a compensatory mechanism to regulate pathological inflammation and restore homeostasis [36]. However, it was negatively correlated with *Kif5b*, which encodes the anterograde kinesin protein, across all *5xFAD* mouse samples except in the HPC of 9-month-old animals. Although initially proposed as an adaptive response to the impaired axonal transport in AD [127], miR-146a-5p was later suggested to be a tauopathy inducer [128]. Additionally, miR-146-5p positively correlated with *Cxcl10* in 6-month-old *5xFAD* mice in the HPC and at 9 months in the PFC. Notably, upregulation of *Cxcl10* in this mouse model was found to be associated with neuroimmune axis dysfunction [129].

All three miRNAs (miR-146a, miR-155-5p, and miR-125b-5p) participate in the regulation of innate immunity and inflammation [130] and were proposed as potential non-invasive tools for identifying individuals at higher risk for AD [131,132]. miR-155-5p is recognized as a pro-inflammatory miRNA that directly inhibits SOCS-1, a negative regulator of cytokine production [40,133]. Our data suggest that miR-125b-5p is positively correlated with miR-155-5p/miR-146a-5p/miR-21-5p in the HPC, while miR-155-5p shows direct association with miR-146a-5p/miR-21-5p in the PFC of 9-month-old *5xFAD* mice.

There are conflicting reports regarding miR-124 dysregulation in AD, with some studies indicating increased levels [22,30,134], and others showing reductions [32,135] in AD patients and models. In our study, we only observed a trend towards upregulated levels when compared to *WT* samples in 9-month-old-mice. Despite these controversies, miR-124 has been linked to neuroprotective and neuroreparative mechanisms [30,31,32]. Results suggest the existence of positive correlations of miR-124-3p with miR-155-5p, miR-146a-5p, and miR-21 in the PFC and with miR-146a-5p, miR-21-5p, and miR-125b-5p in the HPC of 9-month-old *WT* mice. In *5xFAD* mice matched for age, correlations of miR-124-3p with miR-21-5p and miR-125-5p were sustained, but exclusively in HPC.

Evidence indicates that miRNAs often cluster together, exhibiting coordinated expression and collaborative roles in regulating various cellular processes [136]. Our bioinformatic and multivariate statistical analyses align with the regional progression of AD pathology, consistent with recent findings from brain proteome studies in *5xFAD* mice, which revealed the hippocampus to be more profoundly affected by transgene expression than the cortex [46].

Our findings point towards region-specific neuronal and glial transcriptomic profiles, as recently reported by a study analyzing single-cell transcriptomics in six different brain regions from AD patients [137]. Notably, the assessed inflammatory hit miRNAs clustered together in the same branch in PFC, indicating a robust co-expression pattern. This cluster seems to be closely associated with another branch containing the genes *Gfap*, *Trem2*, and *Cst7*, which are consistently upregulated in the *5xFAD* model, suggesting a significant crosstalk between AD-related genes and miRNAs.

Our data collectively suggest that: (1) age significantly influences the expression of genes related to AD onset and progression; (2) *Gfap*, *Cst7*, and *Trem2* are the most promising biomarkers in both the PFC and the HPC from *5xFAD* mice; (3) dysregulation of inflammatory miRNAs and genes is more pronounced at 9 months of age in the *5xFAD* mice; (4) correlations between gene and miRNA expression levels suggest dynamic specificity dependent on genotype, age, and region.

### Limitations

Despite the promising results of our study, there are several limitations that should be acknowledged. Firstly, the present study has limitations related to the constrained number of samples that were evaluated, mainly for animals aged 6 months. Secondly, the predominance of females over male mice may confer a bias to the study. Future studies should confirm the present findings and address this study’s limitations by performing the assessments in a more representative number of samples, by determining sex dependence on the gene and miRNA profiles in the *5xFAD* vs. *WT* mice, and by extending determinations to other biological fluids, such as the blood. Further research using cells derived from AD patients is warranted to validate our findings and facilitate the development of therapeutic options for people suffering from the disease. In future work, more refined techniques, such as single-cell RNA sequencing or spatial transcriptomics, will be necessary to deepen our knowledge about the gene expression patterns in a cell-type-specific manner. Furthermore, expanding the range of molecular targets in our pre-selection of genes would improve the depth of our analysis and potentially will reveal additional pathways involved in disease mechanisms. Lastly, another limitation was the lack of specific miRNA modulation studies in both *WT* and *5xFAD* animals. Conducting such experiments, followed by validation through target analysis and confirmation via western blot, would offer deeper insights into the role of miRNAs in AD.

## 4. Materials and Methods

### 4.1. Animals

This study was performed in the *5xFAD* transgenic mouse line (B6SJL-Tg (APPSwFlLon, PSEN1 * M146L * L286V) 6799Vas/Mmjax; JAX MMRRC Strain #034840) and in their corresponding *WT* littermates (B6SJLF1/J) (at 6 and 9 months of age). These two ages were selected based on previous studies [138,139,140,141], as well as on our interest in defining the best age to be used in future preclinical studies using the miRNA-loaded exosome strategy [114,142]. The model develops amyloid pathology with plaques spreading throughout the hippocampus and cortex by 6 months, together with robust microgliosis and inflammatory processes, as well as behavioral deficits emerging around 4–9 months [19] and was proposed for preclinical testing applications [114].

The *5xFAD* mice bear five AD-linked mutations, which are the Swedish (*K670N/M671L*), Florida (*I719V*), and London (*V717I*) mutations in the APP (isoform 695) transgene, while the PSEN1 transgene harbors the *M146L* and *L286V* ones [19]. The mouse line was maintained at the Rodent Facility at the Instituto de Medicina Molecular João Lobo Antunes, Faculty of Medicine at the University of Lisbon, by crossing the hemizygous *5xFAD* mice with their *WT* background breeders, the B6SJLF1/J, used as controls. All experimental work was carried out minding the 3 Rs in animal research ethics: replacement, reduction, and refinement—minimizing the number of animals necessary. Animals were kept in a controlled temperature (22–24 °C) and humidity (45–65%) room with a 14 h/10 h light/dark cycle and access to food and water ad libitum. Daily surveillance by the Rodent Facility caretakers, researchers, and in-house veterinarian was also performed. All procedures were approved by the Internal Ethics Committee of the Instituto de Medicina Molecular João Lobo Antunes, Faculty of Medicine at the University of Lisbon, and carried out according to the Portuguese law for animal care (Decree-Law No. 113/2013) and the European Community Guidelines for Animal Care (European Union Council Directive 2010/63/EU).

### 4.2. Mouse Brain Isolation and Dissection

*WT* (*n* = 4, all females) and *5xFAD* (*n* = 3, all females) with 6- and 9-month-old mice (*WT*, *n* = 9, 5 males and 4 females; *5xFAD*, *n* = 7, 2 males and 5 females) were sacrificed with an intraperitoneal lethal dose of pentobarbital and decapitated. The brain was quickly removed, and upon isolation, samples were snap frozen in liquid nitrogen and stored at −80 °C until further analysis. Brains from non-perfused *WT* and *5xFAD* mice were defrosted and dissected under a Leica EZ4 Stereo Microscope (Leica Microsystems, Tokyo, Japan), to collect the PFC and HPC regions.

### 4.3. RNA Extraction

The brain tissue was homogenized in TRIzol Reagent (I Thermo Fisher Scientific, Waltham, MA, USA) using a pestle, following the manufacturer’s protocol. Briefly, the TRIzol solution containing lysed samples was mixed with 1:5 volume of chloroform and mixed thoroughly. Then, samples were incubated for 3 min at room temperature and centrifuged at 12,000× *g* for 15 min at 4 °C. The RNA-containing aqueous phase was collected into a new tube, mixed in a 1:2 ratio (referring to the starting volume of TRIzol) with isopropanol to precipitate RNA, and ultimately put overnight at −20 °C, a passage specific for microRNAs. Then, samples were centrifuged at 12,000× *g* for 15 min at 4 °C to pellet RNAs, and the isopropanol was discarded. Pellets were washed with 70% ethanol and centrifuged at 12,000× *g* for 15 min at 4 °C. Finally, the ethanol was discarded, and samples were allowed to dry for at least 20 min at room temperature and resuspended in nuclease-free water. RNA concentration and purity were measured with a NanoDrop Spectrophotometer (Thermo Fisher Scientific). 

### 4.4. Reverse Transcription and RT-qPCR for miRNAs

The miRCURY LNA RT Kit (QIAGEN, Hilden, Germany) was used for cDNA synthesis, and reactions were performed following the manufacturer’s instructions. The final total RNA concentration for the retro-transcription was 1 ng/μL. The reaction had two steps: 42 °C for 60 min, followed by 95 °C for 5 min. The resulting cDNAs were used as templates for RT-qPCR using Power SYBR Green PCR Master Mix (Applied Biosystems, Thermo Fisher Scientific. The PCRs were run in a QuantStudio 7 Flex Real-Time PCR System (Thermo Fisher Scientific, following the program: 95 °C for 10 min, followed by 50 cycles of 95 °C for 10 s and 60 °C for 1 min. The miRNAs analyzed by RT-qPCR are shown in Supplementary Table S5. To verify the specificity of the amplified products, melting curve analysis was performed (ramp from 60 °C to 95 °C, rising 1.6 °C/s), and quantification was performed using the Delta Delta Ct (ΔΔCt) method [143].

### 4.5. Reverse Transcription and RT-qPCR for Genes

Xpert cDNA Synthesis SuperMix (GRiSP, Porto, Portugal) was used for cDNA synthesis, and reactions were performed following the manufacturer’s instructions. The final RNA concentration for the retro-transcription was 50 ng/μL. The resulting cDNAs were used as templates for RT-qPCR using XPert Fast SYBR 2X MasterMix (GRiSP). The PCRs were run in a QuantStudio 7 Flex System. Primers were either designed with PrimerBlast (NCBI) or selected from PrimerBank. Actin was used as internal control (housekeeping gene). The final concentration for each primer was 250 nM. The genes analyzed by RT-qPCR are shown in Supplementary Table S6. The PCR program was as follows: initial step increasing temperature up to 95 °C, incrementing 1.9 °C/s, then holding for 2 min at 95 °C, followed by 50 cycles (95 °C for 5 s and 62 °C for 30 s). To verify the specificity of the amplified products, melting curve analysis was accomplished, and quantification was performed using the above-mentioned Delta Delta Ct (ΔΔCt) method.

### 4.6. Bioinformatic Analyses

R package *multiMiR* (v 1.14.0) [144] was used to search gene targets for our selected miRNAs in the databases miRDB, TarBase, and miR-TarBase. For each miRNA, a gene set including all validated gene targets was constructed. To extract the biological significance of the gene target sets for each specific miRNA, a gene ontology (GO) enrichment analysis was accomplished. To that end, we used the package c*lusterProfiler* (v 4.0.5) [145] to construct biological processes (BP) ontologies. The top 20 GO:BP terms were plotted using SRplot [146].

### 4.7. miRNA–Targeting Network

To create the targeting network, the official miRBase identifiers (IDs) for the mouse (*Mus musculus*) were interrogated on the online platform https://www.mirnet.ca/ (accessed on 24 June 2024). This tool is an open-source platform that comprises eleven miRNA–target prediction databases, including miRTarBase, TarBase, miRecords, SM2mir, Pharmaco-mir, mir2Disease, PhenomiR, StarBase, Epimir, miRDB, and miRanda, mainly focusing on miRNA–target interactions. After setting both the degree and betweenness to 1.0 in the interaction table from the Network Builder menu, the miRNA–target network was generated in the Network Viewer menu (Supplementary Table S2). The analysis revealed a total of 156 nodes (between all our pre-selected genes/miRNAs [33 hits highlighted]), but also another 123 nodes with additional genes/miRNAs in the database (Supplementary Table S3).

### 4.8. Statistical Analyses

GraphPad Prism Software Version 10 (Dotmatics) and RStudio (R Foundation, 2023; https://www.r-project.org/; v 2023.03.1+446) were used to perform statistical analyses and to plot the data. Normality Test was applied for data distribution, and potential outliers were detected and likely removed using Rout Test (Q = 1%). Data are shown as mean ± standard error of the mean (SEM). Statistical differences between groups were defined by a three-way analysis of variance (ANOVA), followed by the Šídák multiple comparisons test. A two-way ANOVA was used to determine statistical differences between the means of three independent groups that have been split by two factors. A *p* value < 0.05 was considered statistically significant. For correlation analyses, variables were centered and standardized prior to analysis to prevent biases due to different scales and dimensions (Supplementary Table S7). Bivariate Pearson’s correlation coefficients were calculated using the cor function, and the resulting correlation matrices were represented using the corrplot package (v 0.92).

## 5. Conclusions

This study elucidated the influence of age, genotype, and brain region sources of variation in inflammation-associated mRNA-miRNA interactions using the *5xFAD* mouse model of AD. Our findings provide valuable insights into the complex interplay between inflammation and neurodegeneration during AD progression, potentially aiding in disease stratification and the development of miRNA-based therapeutics. By targeting miRNAs with broad effects on neurons and glial cells, these therapies might pave the way for novel neuroprotective and neuroregenerative interventions aimed at preventing and/or restoring paracrine cell signaling. Ultimately, our current and future research, by identifying new targets and developing miRNA-based therapeutic strategies, aims to bring hope to millions of individuals suffering from the devastating consequences of AD.

## Data Availability

Available from the corresponding author upon reasonable request.

## Supplementary Materials

The supporting information can be downloaded at: https://www.mdpi.com/article/10.3390/ijms25179475/s1 and https://zenodo.org/records/13355863.
