# Supplementary material for: Spatiotemporal Dysregulation of Neuron–Glia Related Genes and Pro-/Anti-Inflammatory miRNAs in the 5xFAD Mouse Model of Alzheimer’s Disease
Source: Int J Mol Sci. 2024 Aug 31;25(17):9475. doi: 10.3390/ijms25179475 (PMC11394861; doi:10.3390/ijms25179475)
Supplement: Supplementary file 1 [file ijms-25-09475-s001.zip › ijms-3129999-supplementary.pdf]

International Journal of
Molecular Sciences

MDPI

SUPPLEMENTARY DATA

Spatiotemporal dysregulation of neuron-glia related genes and pro-/anti-inflammatory miRNAs in the *5xFAD* mouse model of Alzheimer's disease

International Journal of Molecular Sciences

Marta Ianni [1,2†], Miriam Corraliza-Gomez [1,3,4†], Tiago Costa-Coelho [1,5,6,7], Mafalda Ferreira-Manso [1,5,6,7], Sara Inteiro Oliveira [5,6], Nuno Alemãn-Serrano [5-7], Ana Maria Sebastião [5,6], Gonçalo Garcia [1,8], Maria José Diógenes [5,6], Dora Brites [1,*]

[1] Instituto de Investigação do Medicamento (iMed.ULisboa), Faculdade de Farmácia da Universidade de Lisboa, Portugal; marta.ianni@studenti.unicam.it (M.I.); miriam.corraliza@gm.uca.es (M.C.-G); tcoelho@medicina.ulisboa.pt (T.C.); mafalda.manso@medicina.ulisboa.pt (M.M.); ggarcia@campus.ul.pt (G.G.); dbritestes@ff.ulisboa.pt (D.B.)
[2] Università degli Studi di Trieste, Dipartimento di Scienze della Vita, Trieste, Italy;
[3] Universidad de Cadiz, Division of Physiology, School of Medicine, Cadiz, Spain;
[4] Instituto de Investigación e Innovación Biomédica de Cadiz (INIBICA), Cadiz, Spain;
[5] Instituto de Farmacologia e Neurociências, Faculdade de Medicina da Universidade de Lisboa, Portugal; sara86oliveira@medicina.ulisboa.pt (S.I.O.; nuno.aleman@campus.ul.pt (N.A.); anaseb@medicina.ulisboa.pt (A.S.); diogenes@medicina.ulisboa.pt (M.J.D.)
[6] Instituto de Medicina Molecular João Lobo Antunes, Faculdade de Medicina da Universidade de Lisboa, Portugal;
[7] ULS Santa Maria, Centro Hospitalar Universitário Lisboa Norte, Centro Académico de Medicina de Lisboa, Portugal;
[8] Department of Pharmaceutical Sciences and Medicines, Faculdade de Farmácia da Universidade de Lisboa, Portugal;

† These authors contributed equally to this work.
* Correspondence: dbrites@ff.ulisboa.pt

**Supplementary Table S1**	Gene expression profile by RT-qPCR analysis revealed no significant differences when simultaneously considering the genotype (WT/5xFAD), age (6/9 months) and brain region (HPC, hippocampus/PFC, prefrontal cortex).	Link
**Supplementary Table S2**	miRNA-target table for the Analyzed microRNAs and targets selected for this study. Obtained in the online platform https://www.mirnet.ca/	Link
**Supplementary Table S3**	Node table for the analyzed microRNAs and targets selected for this study. We only considered miRNAs and/or targets with a node degree of at least 2. Obtained in the online platform https://www.mirnet.ca/	Link
**Supplementary Table S4**	Bivariate Pearson's correlation coefficients and respective *p*-values obtained between all miRNAs and genes.	Link
**Supplementary Table S5**	List of microRNAs analyzed by RT-qPCR and their primer sequences.	Link
**Supplementary Table S6**	List of genes and respective primer sequences used for mRNA analysis by RT-qPCR.	Link
**Supplementary Table S7**	Raw data used for correlational analysis in hippocampus (HPC) and prefrontal cortex (PFC) using the corrplot package in RStudio software.	Link
