# Supplementary material for: Spatiotemporal Dysregulation of Neuron–Glia Related Genes and Pro-/Anti-Inflammatory miRNAs in the 5xFAD Mouse Model of Alzheimer’s Disease
Source: Int J Mol Sci. 2024 Aug 31;25(17):9475. doi: 10.3390/ijms25179475 (PMC11394861; doi:10.3390/ijms25179475)
Supplement: Supplementary file 1 [file ijms-25-09475-s001.zip › Supplementary Table S5_microRNA primers.pdf]

Supplementary Table S5. List of microRNAs analyzed by RT-qPCR and their primer sequences.

microRNA	Target Sequence (5'-3')
**hsa-miR-124-3p**	UAAGGCACGCGGUGAAUGCC
**hsa-miR-146a-5p**	UGAGAACUGAAUUCCAUGGGUU
**hsa-miR-125b-5p**	UCCCUGAGACCCUAACUUGUGA
**hsa-miR-21-5p**	UAGCUUAUCAGACUGAUGUUGA
**mmu-miR-155-5p**	UUAAUGCUAAUUGUGAUAGGGGU
**mmu-SNORD110**	Reference Gene
