# Supplementary material for: Spatiotemporal Dysregulation of Neuron–Glia Related Genes and Pro-/Anti-Inflammatory miRNAs in the 5xFAD Mouse Model of Alzheimer’s Disease
Source: Int J Mol Sci. 2024 Aug 31;25(17):9475. doi: 10.3390/ijms25179475 (PMC11394861; doi:10.3390/ijms25179475)
Supplement: Supplementary file 1 [file ijms-25-09475-s001.zip › Supplementary Table S6_transcripts primers.pdf]

Supplementary Table S6. List of genes and respective primer sequences used for mRNA analysis by RT-qPCR.

Gene	Species	Primer Sequence (5' - 3')	Amplicon Size
B-actin	mmu	F: GCAGGAGTACGATGAGTCCG<br>R: ACGCAGCTCAGTAACAGTCC	74
Rbfox3 – NeuN	mmu	F: CCAGGCACTGAGGCCAGCACACAGC<br>R: CTCCGTGGGGTCGGAAGGGTGG	111
Dlg4 – PSD95	mmu	F: GACGCCAGCGACGAAGAG<br>R: CTCGACCCGCCGTTTG	96
Cx3cl1	mmu	F: CTCACGAATCCCAGTGGCTT<br>R: TTTCTCCTTCGGGTCAGCAC	121
Dnah1 – Dynein	mmu	F: CGCCTGTCTTCTGGATCAGT<br>R: ATTTCTGATGACGCCTCGGG	142
Kif5b – Kinesin	mmu	F: GGCGGAGTGCAACATCAAAG<br>R: TGCTTGACTGGAACACACGA	155
Plp1	mmu	F: TGGCGACTACAAGACCACCA<br>R: GACACACCCGCTCCAAAGAA	116
P2ry1	mmu	F: GAGGTGCCTTGGTCGGTTG<br>R: CGGCAGGTAGTAGAACTGGAA	159
Gfap	mmu	F: CAAACTGGCTGATGTCTACC<br>R: GCTTCATCTGCCTCCTGTCTA	207
Gja1 – Connexin43	mmu	F: CTGAGTGCGGTCTACACCTG<br>R: GAGCGAGAGACACCAAGGAC	127
Nfia	mmu	F: TTCCAACGTCACCCATCATCC<br>R: CAGCATCAGGACAGACAAGTT	114
Cxcr3	mmu	F: TCACAGCCCTGTCCCAA<br>R: CGTCTCCCCCAGTGCTTCAA	90
Trem2	mmu	F: CTGATCACAGCCCTGTCCCAA<br>R: CGTCTCCCCCAGTGCTTCAA	110
Arg1	mmu	F: CTTGGCTTGCTTCGGAACTC<br>R: GGAGAAGGCGTTTGCTTAGTTC	146
Tlr4	mmu	F: ACCTGGCTGGTTTACACGTC<br>R: GTGCCAGAGACATTGCAGAA	201
H2Aa – MhcII	mmu	F: TGGGCACCATCTTCATCATTC<br>R: GGTCACCCAGCACACCACTT	131
Ccr7	mmu	F: CATGGACCCAGGTGTGCTTC<br>R: TCAGTATCACCAGCCCGTTG	195
P2ry12	mmu	F: GCAGAACCAGGACCATGGAT<br>R: CTGACGCACAGGGTGCTG	88
Cst7	mmu	F: GGAGCTGTACTTGCCGAGC<br>R: CATGGGTGTCAGAAGTTAGGC	108
Mfn2	mmu	F: CAGAGCAGAGCCAAACTGCT<br>R: AACATGTTGAGTTCGCTGTCC	62
Dnm1l - Drp1	mmu	F: GCTCAGTGCTGGAAAGCCTA<br>R: TCCATGTGGCAGGGTCATTT	150
Bcl2	mmu	F: ATGCCTTTGTGGAACTATATGGC<br>R: GGTATGCACCCAGAGTGATGC	120
Cxcl10	mmu	F: CCAAGTGCTGCCGTCATTTTC<br>R: GGCTCGCAGGGATGATTTCAA	157
Il18	mmu	F: GACTCTTGCGTCAACTTCAAGG<br>R: CAGGCTGTCTTTTGTCAACGA	169
Tnfa	mmu	F: CATCTTCTCAAAATTCGAGTGACAA<br>R: TGGGAGTAGACAAGGTACAACCC	175

Mfge8	mmu	F: CAATGAGACTGAGAGAGAGGACCAT<br>R: CCATCCAGGTTGTAGTAGTTGGT	176
Traf6	mmu	F: AAAGCGAGAGATTCTTTCCCTG<br>R: ACTGGGGACAATTCACTAGAGC	125
Cebpa	mmu	F: AGCTTACAACAGGCCAGGTTTC<br>R: CGGCTGGCGACATACAGTAC	192
Irak1	mmu	F: GAGACCCTTGCTGGTCAGAG<br>R: GCTACACCCACCCACAGAGT	137
Socs1	mmu	F: CTGCGGCTTCTATTGGGGAC<br>R: AAAAGGCAGTCGAAGGTCTCG	216
